# Meals from community programs and senior centers in older US adults – at risk of becoming a relic of the past?

**DOI:** 10.1016/j.jarlif.2026.100078

**Published:** 2026-07-09

**Authors:** Maximilian Andreas Storz, Ruopeng An

**Affiliations:** a Department of Internal Medicine II, Centre for Complementary Medicine, Medical Center – University of Freiburg, Faculty of Medicine, University of Freiburg, Freiburg, Germany; b Silver School of Social Work, New York University, NY, NY, United States

**Keywords:** Community center, Senior center, Nutrition programs, Aging, Public health

## Abstract

**Background:**

Community and senior centers are focal points of community-based services. Despite their pivotal role in the aging continuum of care and in light of anticipated budgetary cuts impacting the Older Americans Act, their relevance is suggested to decrease in the US.

**Methods:**

Using NHANES (National Health and Nutrition Examination Survey) data from 2009 to 2023, we described national secular trends in the proportion of US adults aged ≥60 years reporting community/senior center meals (CSCMs). Sociodemographic factors associated with CSCMs consumption were identified using multivariable logistic regression.

**Results:**

Data from 514 participants eating CSCMs were analyzed. The proportion of individuals reporting CSCMs ranged from 6.4% (in 2009–2010) to 2.4% (in 2021–2023). Participants reporting CSCMs were predominantly female (64.43%), widowed/divorced (47%), and had an annual income <20,000$ (35.85%). In comparison to males, females who did not complete the 9th grade and female college graduates were less likely to eat CSCMs (Odds Ratios (OR): 0.31 (0.18–0.55) and 0.30 (0.14–0.66), respectively; *P* = 0.001).

**Conclusions:**

>2% of the older population currently obtains CSCMs. Given their participation-based reimbursement model, community and senior centers offering meals could soon become a relic of the past. Public health strategies may consider the herein presented participation predictors to tailor individualized programs, increasing attendance.

## Highlights

• In 2009–2010, 6.4% of US adults ≥60 years reported senior/community center meals.
• In 2021–2023, 2.4% of US adults ≥60 years reported senior/community center meals.
• A national secular trend analysis suggested a substantial decline over time.
• Age, income and food security were associated with senior/community center meals.
• The interaction of sex and education was associated with senior/community center meals.

BODY TEXT

## 1 Introduction

Senior and community centers are focal points of assistance and services centering around nutrition, health and fitness, recreational activities, volunteering opportunities, and social support [1,2]. Despite a growing population of older adults in the US and despite their pivotal role in community-based services in the aging continuum of care, the relevance of senior and community centers has been suggested to decrease in the US [1,3].

Senior and community centers are reimbursed based on participation rates, and a decrease in attendance may thus impact them negatively [1]. Operating senior and community centers is a financial challenge, and given increasing expenses, policymakers and public service administrators are often interested in learning who attends senior and community centers and why [1]. Understanding the rationale and needs of senior and community center participants enables administrators to tailor individualized recruitment strategies and allocate public resources more effectively.

While studies conducted over the last decades have identified sociodemographic characteristics associated with senior and community center participation [1], specific information on the nutrition domain (e.g., meal preparation/cooking programs) is still scarce. Previous studies were often state-specific [1], whereas nationally representative samples have been analyzed rarely.

The purpose of this study was to describe secular trends in older U.S. adults obtaining meals from senior and community centers at the national level, using data from the National Health and Nutrition Examination Surveys (NHANES, 2009–2023). A special emphasis was put on sociodemographic factors associated with meals from senior and community centers.

## 2 Methods

### 2.1 Study population

The NHANES is a cross-sectional study designed to provide population-level estimates of the prevalence of health-related outcomes among the civilian US population [4,5]. The NHANES employs a multistage, stratified, clustered, and probability sampling design [4]. The NHANES obtained ethical approval from the National Center for Health Statistics Research Ethics Review Board. The study was conducted in accordance with the Declaration of Helsinki. Six NHANES cycles (2009–2010 through 2017–2018 and 2021–2023) were combined for this secular trend analysis. This work followed the STROBE (Strengthening the Reporting of Observational Studies in Epidemiology) guidelines (see: Supplementary Table 1) [6].

### 2.2 Outcome

The use of prepared meals provided by senior and community centers (hereafter referred to as community/senior center meals) was extracted from the NHANES “Diet Behavior and Nutrition” module [7]. Adults aged ≥60 years were asked: “In the past 12 months, did you go to a community program or senior center to eat prepared meals?”. The weighted proportion of participants reporting community or senior center meals was estimated for each NHANES cycle. The incomplete NHANES pandemic cycle was not considered for this analysis for analytical purposes.

### 2.3 Covariates/predictors

Sociodemographic data included sex (categorical variable: male; female), ethnicity (categorical variable: Mexican American; Other Hispanic; Non-Hispanic White; Non-Hispanic Black; Other Race), education level (categorical variable: <9^th^ grade; 9–11^th^ grade; high school graduate; some college or AA degree; college graduate), marital status (categorical variable: married / with partner; widowed / divorced / separated; never married), household size (continuous predictor: in persons per household), citizenship status (categorical variable: citizen by birth or naturalization; not a citizen of the US), and age (in years). Socioeconomic data included annual household income (categorical variable: < $20,000; ≥ $20,000 & < $75,000; ≥ $75,000), income from retirement or survivor pensions (categorical variable: yes; no), health insurance coverage (categorical variable: yes; no), food security category (categorical variable: full food security; marginal food security; low food security; very low food security), poverty - based on the ratio of family income to poverty level (categorical variable: yes (ratio < 1); no (ratio ≥ 1)), and expenses at supermarkets or grocery stores (in $).

### 2.4 Statistical analysis

The analysis was performed in Stata 18 (StataCorp. 2023. Stata Statistical Software: Release 18. TX: StataCorp LLC.); Stata’s “. svyset” and “. svy” commands were used to account for the NHANES survey design characteristics. A 12-year sample weight for household interview data was constructed for the secular trend analysis (part I). Part II, which focused on sociodemographic factors associated with community/senior center meals consumption, relied on the NHANES cycles from 2009 to 2018.

Data from participants who reported eating meals at senior and community centers and those who denied doing so were compared using unconditional subclass analysis [8]. Categorical variables were described with their weighted proportions and 95%-confidence intervals (CI). Data presentation standards for weighted proportions were considered [8,9].

Associations between sociodemographic variables and meal usage at community and senior centers were examined using the Rao-Scott F-test. Between-group differences were compared using Stata’s lincom command (weighted proportions) or adjusted Wald tests (normally distributed continuous variables). Logistic regression models predicting the likelihood of reporting community or senior center meals were built based on the survey analysis techniques described by Heeringa et al. [10]. All variables were entered as categorical variables into the regression model, except for age, household size, and supermarket expenses. Odds Ratios (OR) from the multivariable logistic regression model were graphically visualized in a coefficient plot [11].

## 3 Results

**Fig. 1:**
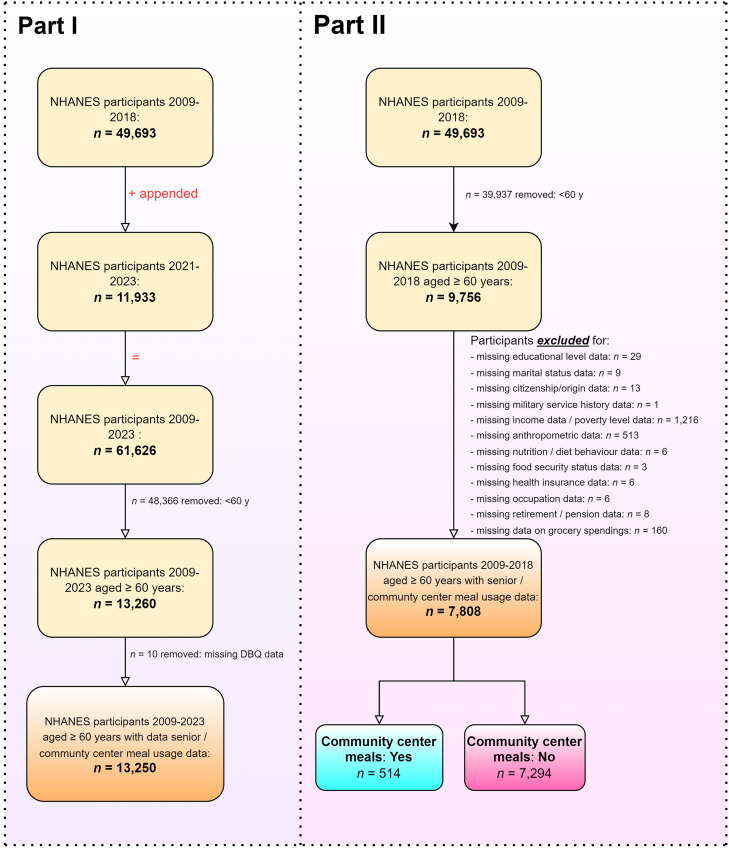
Participant inclusion flowchart with detailed reasons for inclusion and exclusion of participants.

**Fig. 2:**
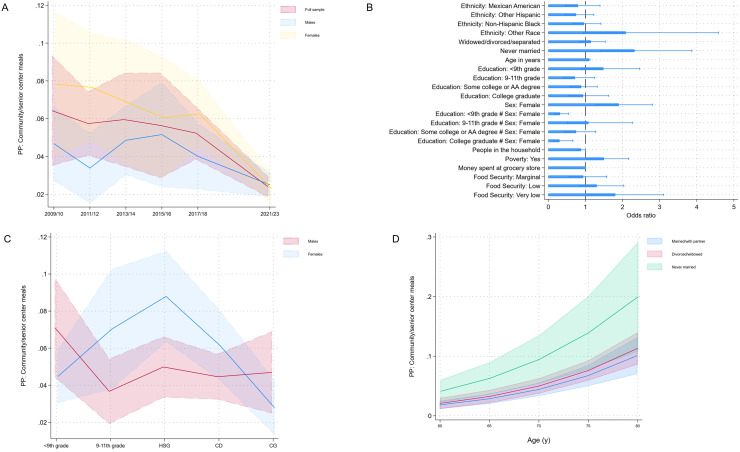
Secular trends in community programm-/ senior-center meal consumption Fig. 2: Based on n = 7808 unweighted observations. Panel A depicts the secular trend analysis (part I). Panel B visualizes the coefficients of the employed multivariable regression model (part II). Panel C illustrates marginal predicted probabilities for meals from senior and community centers depending on a sex-education interaction. Significant predicted probabilities were found for men and women who completed the 9th grade and for those who finished high school. Panel D displays marginal predicted probabilities for meals from senior and community centers, depending on marital status and age. Never-married participants were 2.32 times more likely to eat meals at community or senior centers (CI: 1.39–3.87, *P=0.002*).

Part I – the secular trend analysis - was based on 13,250 participants. Part II included 7808 participants, of whom 514 individuals reported eating meals at community or senior centers. Fig. 1 displays a participant inclusion flowchart for both parts. Fig. 2 shows the predicted probability for a participant reporting community/senior center meals for each NHANES cycle, which ranged from 0.064 (in 2009–2010) to 0.024 (in 2021–2023). This contrast was statistically significant (*P* = 0.008).

Table 1 displays the sample’s characteristics (part II). The community/senior center meal subpopulation was predominantly female with a mean age of 73.58 years. Community/senior center meals were associated with educational level and marital status. More than one-third of this subpopulation indicated an annual household income of ≤$20,000.

**Table 1:** Sample characteristics by community program-/ senior-center meal consumption.

	*Community program-/ senior-center meals: Yes* *(n=7294)*	*Community program-/ senior-center meals: No* *(n=514)*	*P-value*
**Sex**			*p* < 0.001 ^a^
Male	46.05% (44.84–47.26)	35.57% (30.30–42.21) ^⁎^	
Female	53.95% (52.74–55.16)	64.43% (58.79–69.70) ^⁎^	
**Age (years)**	69.13 (68.87–69.40)	73.58 (72.61–74.56)	*p* < 0.001 ^b^
**Ethnicity/race**			*p* = 0.138 ^a^
Mexican American	3.99% (2.95–5.36)	2.91% (1.71–4.91)	
Other Hispanic	3.53% (2.76–4.50)	2.49% (1.58–3.90)	
Non-Hispanic White	78.10% (75.44–80.55)	75.25% (67.23–81.83)	
Non-Hispanic Black	8.42% (7.13–9.92)	9.80% (6.99–13.57)	
Other Race ^a^	5.96% (5.02–7.09)	9.56% (5.08–17.27) ^✝^	
**Education level**			*p* < 0.001 ^a^
<9^th^ grade	6.69% (5.77–7.74)	9.20% (6.93–12.12)	
9–11^th^ grade	9.58% (8.43–10.88)	12.45% (8.42–18.02)	
High school graduate/GED	23.82% (22.06–25.67)	33.40% (28.22–39.03) ^⁎^	
Some college or AA degree	29.74% (27.80–31.75)	28.41% (24.44–32.74)	
College graduate or above	30.18% (27.58–32.91)	16.54% (12.21–22.03) *	
**Marital status**			*p* < 0.001 ^a^
Married or living with a partner	65.67% (63.80–67.49)	46.10% (40.24–52.06) ^⁎^	
Divorced/separated/widowed	30.59% (28.83–32.41)	47.02% (40.73–53.40) ^⁎^	
Never married	3.74% (3.23–4.33)	6.89% (4.77–9.85) ^⁎^	
**Born in the United States**			*p* = 0.227 ^a^
Yes	87.61% (85.92–89.12)	89.17% (86.06–91.65)	
No	12.39% (10.88–14.08)	10.83% (8.35–13.94)	
**Citizenship status**			*p* = 0.320 ^a^
US citizen by birth or naturalization	96.81% (96.19–97.29)	97.76% (95.40–98.93)	
Not a citizen of the US	3.19% (2.71–3.76)	2.24% (1.07–4.60) ^✝^	
**Served in the US Armed Forces**			*p* = 0.327 ^a^
No	79.26% (77.81–80.64)	81.08% (77.62–84.12)	
Yes	20.74% (19.36–22.19)	18.92% (15.88–22.38)	
**Ratio of family income to poverty**			*p* < 0.001 ^a^
< 1 (below poverty threshold)	9.59% (8.42–10.89)	18.03% (13.93–23.02) ^⁎^	
≥ 1 (above poverty threshold)	90.41% (88.11–91.58)	81.97% (76.98–86.07) ^⁎^	
**Annual household income**			*p* < 0.001 ^a^
< $20,000	15.24% (13.60–17.03)	35.85% (30.81–41.23) ^⁎^	
≥ $20,000 & < $75,000	52.08% (49.55–54.60)	48.32% (42.08–54.61)	
≥ $75,000	32.68% (29.84–35.66)	15.83% (11.06–22.12) ^⁎^	
**Adult food security category**			*p* = 0.029 ^a^
Full food security	84.68% (83.04–86.19)	79.97% (74.88–84.25) ^⁎^	
Marginal food security	6.21% (5.37–7.18)	6.18% (3.90–9.68)	
Low food security	5.15% (4.47–5.93)	6.89% (4.97–9.48)	
Very low food security	3.96% (3.20–4.87)	6.95% (4.52–10.55) ^⁎^	
**Money spent at supermarket/grocery store**	386.30 (367.30–405.31)	264.40 (235.75–293.08)	*p* < 0.001 ^b^
**Number of people in the household**	2.19 (2.14–2.23)	1.83 (1.72–1.95)	*p* < 0.001 ^b^
**Covered by health insurance?**			*p* = 0.020 ^a^
Yes	95.16% (94.30–95.90)	97.55% (95.67–98.62) ^⁎^	
No	4.84% (4.10–5.70)	2.45% (1.38–4.32) ^⁎^	
**Income from retirement/survivor pension**			*p* = 0.810 ^a^
Yes	41.36% (39.10–43.66)	42.12% (35.62–48.90)	
No	58.64% (56.34–60.90)	57.88% (51.10–64.38)	
**Self-perceived diet quality/health**			*p* = 0.079 ^a^
Excellent or very good	41.37% (39.50–43.26)	37.00% (31.83–42.48)	
Good or fair	55.38% (53.59–57.16)	57.88% (52.58–63.01)	
Poor	3.25% (2.71–3.90)	5.11% (3.32–7.81)	
**Type of work done last week**			*p* < 0.001 ^a^
Working at a job or business	30.75% (29.19–32.37)	15.89% (10.63–23.08) ^⁎^	
Looking for work	1.02% (0.76–1.38)	0.29% (0.06–1.35) ^*,✝^	
Not working at a job or business	68.22% (66.57–69.84)	83.82% (76.64–89.11) ^⁎^	
**BMI (kg/m²)**	29.42 (29.16–29.67)	28.86 (28.13–29.59)	*p* < 0.137 ^b^

Table 1 shows weighted proportions. The underlying total number of unweighted observations was N = 7808. Continuous variables are presented as the mean (95% confidence interval). Categorical variables are presented as weighted proportions (95% confidence interval). All weighted proportions can be considered reliable, as per the recent guidelines from the National Center for Health Statistics, except for those marked with a ^“†”^ symbol, which denotes an unreliable proportion. ^a^ = based on Stata’s design-adjusted Rao-Scott test, ^b^ = based on regression analyses followed by adjusted Wald tests. The ^“*”^ symbol denotes significant between-group differences in the weighted proportions.

Logistic regression was used to determine the effects of race/ethnicity, marital status, age, education level, sex, household size, poverty level, food security, and grocery store expenses on the likelihood of eating meals from senior and community centers. The complete model including reference categories for each variable is listed in Table 2.

**Table 2:** Survey-weighted logistic regression model ascertaining the effects of various sociodemographic predictors on the likelihood of eating meals from senior and community centers (NHANES 2009–2018; *N* = 7808).

Variables	OR	95%-CI	*P*-value
***Race/ethnicity***			
Mexican American	0.80	0.46 – 1.39	0.425
Other Hispanic	0.75	0.46 – 1.23	0.245
Non-Hispanic White	*REF*	-	-
Non-Hispanic Black	0.96	0.65 – 1.42	0.828
Other Race	2.09	0.95 – 4.59	0.067
***Marital status***			
Married / with partner	*REF*	-	-
Widowed/divorced/separated	1.14	0.85 – 1.53	0.363
Never married	2.32	1.39 – 3.87	0.002
***Age (in years)***	1.10	1.07 – 1.12	<0.001
***Educational level***			
<9th grade	1.48	0.89 – 2.46	0.126
9–11th grade	0.72	0.41 – 1.25	0.235
High school graduate	*REF*	-	-
Some college or AA degree	0.89	0.60 – 1.32	0.542
College graduate	0.94	0.54 – 1.63	0.812
***Sex***			
Male	REF	-	-
Female	1.90	1.28 – 2.81	0.002
***Education#Sex***			
<9th grade#female	0.31	0.18 – 0.55	<0.001
9–11th grade#female	1.08	0.51 – 2.26	0.843
High school graduate#male	*REF*	-	-
Some college or AA degree#female	0.75	0.44 – 1.27	0.280
College graduate#female	0.30	0.14 – 0.66	0.003
***People in the household (n)***	0.88	0.77 – 1.00	0.050
***Poverty***			
No poverty	*REF*	-	-
Poverty	1.50	1.04 – 2.16	0.031
***Money spent in grocery stores ($)***	0.998	0.997 – 0.999	0.003
***Food security level***			
Full	*REF*	-	-
Marginal	0.94	0.56 – 1.57	0.813
Low	1.30	0.84 – 2.03	0.238
Very low	1.80	1.05 – 3.11	0.034

Table 2 The underlying total number of unweighted observations was N = 7808. AA = Associate of Arts degree. CI = Confidence Interval. OR = Odds Ratio. REF = Reference category. All variables were entered as categorical variables into the regression model, except for age (in years), household size (in number of persons), and supermarket expenses (in $).

Fig. 2 visualizes the obtained OR based on this model. To avoid multicollinearity, we omitted annual household income in the final model and considered poverty level instead.

In comparison to males, females who did not complete the 9^th^ grade and college graduates were significantly less likely to eat meals at senior and community centers (OR: 0.31 (0.18–0.55) and 0.30 (0.14–0.66), respectively). Living below the poverty threshold was associated with an increased likelihood of eating at community/senior centers (OR: 1.50 (1.04–2.16), *P* = 0.027) when compared to living above the poverty threshold. Increasing age increased the likelihood of eating at community/senior centers (OR: 1.09 (1.07–1.12) for each 1-year increase, *P* = 0.031), whereas increasing supermarket/grocery expenses decreased the likelihood (OR: 0.998 for each 1-$ increase (0.997–0.999), *P* = 0.003). Participants in the lowest food-security category were 1.80 times more likely to eat meals from senior and community centers (CI: 1.05–3.11, *P* *=* *0.034*) than fully food secure participants. Fig. 2 illustrates the predicted probabilities of reporting community center meals based on the interaction between sex and educational level (see Table 2). Sex-specific contrasts were significantly different for high school graduates and those who completed the 9th grade (*P* *=* *0.001*).

## 4 Discussion

Senior and community centers are an effective model for engaging community-dwelling older adults in health activities; however, their importance in the US has been suggested to decline [1]. To the best of our knowledge, population-based analyses at the national level are scarce in this context. The analyzed data herein suggest a declining trend in senior/community center meals between 2009 and 2023 at the national level, corroborating previously suggested local trends [1].

Several identified predictors associated with an increased likelihood of consuming meals at senior and community centers are consistent with the literature (e.g., women of higher age, low income). Our findings thus reiterate that senior and community centers are of great importance when it comes to socialization and companionship in older adults [1,12]. The identified sex-education interaction could be of importance to program designers and illustrates the challenges of designing innovative programs aimed at attracting a wider audience. The associations between meals from senior and community centers and income, as well as food security, also underscore their essential role in the context of social care and the basic provision of meals to an increasing part of the US population [13].

Our data enable administrators and policymakers to tailor community/senior center meal program recruitment strategies and sustainably allocate public resources in light of a constant demand for meals, as suggested based on nationally representative data.

Senior center directors, policy makers and municipalities may consider the capacity to serve a larger share of the community and subsequently adjust the types of offerings provided. Gearing additional information and recruitment strategies targeted at those underrepresented in participation at present (e.g., food-secure males) could help increase participation and increase revenue [14]. In this context, Somerville et al. suggested to increase the visibility of local centers to “function as an information and support resource in the community” in order to create communities where all older adults can live independent, thereby deferring morbidity and disability as long as possible [14].

Our data, however, indicate a decline in meals from senior and community centers, implying that, if current trends continue, senior and community centers may soon become a relic of the past due to their participation-based reimbursement model. Such a scenario might not only affect nutrition-related outcomes and nutritional resilience but older adults’ functional and social life in a more general way. Senior centers offer numerous services beyond the provision of meals and may support those in need of caregiver education, financial assistance, health and wellness programs, fitness and exercise, library services, social networking and transportation [15]. While not directly assessed in the present study, a decline in the availability of and participation in senior and community center services could thus increase social isolation and increase nutritional vulnerability over time. This scenario is not directly supported by the herein examined data but conceivable given the pivotal role of senior and community centers in influencing and maintaining health and well-being in older adults in the United States [1].

The herein presented results suggest that individuals living below the poverty threshold and in the lowest food-security category use senior center meal services more frequently, thereby emphasizing a potential “safety net” role of these facilities for this particular population. The decline in participation may thus be interpreted beyond a simple trend but could also signal a shift in the performance environment for vulnerable subpopulations of older adults. Senior and community centers have been termed a “gateway to the nation’s aging network” by connecting older US adults to vital community services [16]. A decline in senior and community center infrastructures may thus not only contribute to increased food security over time but hypothetically to an increasing workload and burden for other third sector organizations and community-led volunteering programs designed to support healthy aging in older adults [17]. If the herein presented scenario materializes further, other organizations and stakeholders may have to increase their efforts and resources to buffer the potential impact. With regard to meal provision, this could for instance affect local food banks and pantries, where available.

Study limitations include the lack of geographical data (e.g., rural vs. urban location) and the absence of other potentially relevant predictors (e.g., mental health or alcohol intake). The herein found decline over time has not been reported by Meyer and Heflin, who used data from the Current Population Survey Food Security Supplement and who conducted interviews with participants of such services [18,19].

Nevertheless, our study adds to the literature by re-emphasizing the importance of senior/community center meal programs and by highlighting that >2% of the older US population consistently report meal consumption from these focal care points.

## 5 Conclusions

Between 6.4% (2009–2010) and 2.4% (2021–2023) of the US adult population ≥60 years reported senior/community center meals; a trend analysis suggested a substantial decline over time. These findings have important public health implications, as 2.4% of the older US population still relies on these meals. The decreasing demand for meals at senior/community centers may pose a challenge to these centers as they are reimbursed based on participation rates. Without a change in practice and reimbursement policies, community and senior centers offering meals could soon become a relic of the past. Public health strategies may consider the herein presented participation predictors to tailor individualized programs, increasing attendance.

## Declarations

This work has not been previously published and is not under consideration for publication elsewhere. All co-authors have approved this work.

## Ethical considerations

NHANES was approved by the National Center for Health Statistics' research ethics review board, and informed consent was obtained from all participants. The study was conducted in accordance with the Declaration of Helsinki.

## Consent for publication

The National Center approved NHANES for Health Statistics' research Ethics Review Board, and informed consent was obtained from all participants.

## Data availability

Data is publicly available online (https://wwwn.cdc.gov/nchs/nhanes/Default.aspx). The datasets used and analyzed during the current study are available from the corresponding author on request.

## Funding

The authors received no financial support or funding for this work.

## Declaration of the use of generative AI and AI-assisted technologies in scientific writing and in figures, images and artwork

No AI / AI-assisted technologies were used for the preparation of this manuscript.

## Supplementary data

Supplementary data – Supplementary Table 1.

STROBE Checklist (uploaded as a separate file)

## CRediT authorship contribution statement

**Maximilian Andreas Storz:** Writing – review & editing, Writing – original draft, Visualization, Validation, Software, Resources, Project administration, Methodology, Investigation, Funding acquisition, Formal analysis, Data curation, Conceptualization. **Ruopeng An:** Writing – review & editing, Validation, Supervision, Project administration, Investigation.

## Declaration of competing interest

The authors declare that they have no known competing financial interests or personal relationships that could have appeared to influence the work reported in this paper

## Appendix Supplementary materials

Image, application 1

## References

[1] Pardasani M., Berkman C. (2021). New York City Senior centers: who participates and why?. J Appl Gerontol.

[2] Forbes (2023). What are senior centers and why are they important?. Forbes Health.

[3] Pardasani M. (2019). Senior centers: if you build will they come?. Educ Gerontol.

[4] CDC (2026). National health and nutrition examination survey. Natl Health Nutr Exam Surv.

[5] Yang L., Toriola A.T. (2024). Menopausal hormone therapy use among postmenopausal women. JAMA Health Forum.

[6] Elm von E., Altman D.G., Egger M. (2007). The strengthening the reporting of observational studies in Epidemiology (STROBE) statement: guidelines for reporting observational studies. PLOS Med.

[7] NHANES Diet Behavior & Nutrition module (DBQ_J) (2020). https://wwwn.cdc.gov/Nchs/Data/Nhanes/Public/2017/DataFiles/DBQ_J.htm.

[8] Storz M.A., Ronco A.L. (2024). Diet quality in U.S. adults eating in senior and community centers: NHANES 2009-2018. J Nutr Health Aging.

[9] Parker J.D., Talih M., Malec D.J. (2017). National Center for Health Statistics Data Presentation Standards for proportions. Vital Health Stat 2.

[10] Heeringa S.G., Berglund P.A., Berglund P.A. (2017).

[11] Jann B. (2025). COEFPLOT: stata module to plot regression coefficients and other results. https://EconPapers.repec.org/RePEc:boc:bocode:s457686.

[12] Keyes L., Li Q., Collins B. (2022). Senior center service utilization: do social ties affect participation patterns?. J Appl Gerontol.

[13] USDA (2026). Food Security in the US - key statistics & graphics | Economic Research Service. https://www.ers.usda.gov/topics/food-nutrition-assistance/food-security-in-the-us/key-statistics-graphics.

[14] Somerville C., Roldán N.V., Bui C.N., Coyle C., Mutchler J. (2022). Why don’t older adults use senior centers? Evidence from adults age 50 and older in Massachusetts. J Elder Policy.

[15] Wick J.Y. (2012). Senior centers: traditional and evolving roles. Consult Pharm: J Am Soc Consult Pharm.

[16] National Council on Aging (2024). Get the facts on senior centers and how they serve older adults. https://www.ncoa.org/article/get-the-facts-on-senior-centers/.

[17] Davies R., Reid K. (2024). Supporting each other: older adults’ experiences empowering food security and social inclusion in rural and food desert communities. Appetite.

[18] Meyer M.H., Heflin C.M. (2026). Community-based food program limits in reducing older adult food insecurity Maxwell School. https://www.maxwell.syr.edu/research/lerner-center/population-health-research-brief-series/article/community-based-food-program-limits-in-reducing-older-adult-food-insecurity.

[19] Heflin C.M., Meyer M.H. (2025). Food for thought: understanding older adults food insecurity. Russell Sage Found.

