# Supplementary material for: Meals from community programs and senior centers in older US adults – at risk of becoming a relic of the past?
Source: JAR Life. 2026 Jul 9;15:100078. doi: 10.1016/j.jarlif.2026.100078 (PMC13380230; doi:10.1016/j.jarlif.2026.100078)
Supplement: Supplementary file 1 [file mmc1.doc]

STROBE Statement—Checklist of items that should be included in reports of ***cross-sectional studies***

|  | Item No | Recommendation |
| --- | --- | --- |
| **Title and abstract** | 1 | (*a*) Indicate the study’s design with a commonly used term in the title or the abstract  *-> indicated in the abstract under the subheading “Design”* |
|  |  | (*b*) Provide in the abstract an informative and balanced summary of what was done and what was found  *-> Page 2, abstract emphasizes: that national secular trends in the proportion of US adults reporting community/senior center meals were described. It is further explained that sociodemographic factors associated with community/senior center meal consumption in US adults aged 60 years or older were identified.* |
| Introduction | | |
| Background/rationale | 2 | Explain the scientific background and rationale for the investigation being reported  *-> Page 3 summarizes that senior and community centers are focal points of services, assistance and other programs centering around senior health and wellbeing. Despite a growing population of older adults in the US, and despite their pivotal role as focal points of community-based services in the aging continuum of care, the relevance of senior and community centers decreases in the US. It is further explained that operating these centers is a financial challenge and given the increasing expenses, policy makers and public service administrators are often interested in learning who attends these centers and why. Understanding the rationale and needs of participants allows administrators and policy makers to tailor recruitment strategies and to sustainably allocate public resources. To the best of our knowledge, no study has investigated secular trends in community center meal usage in the US at the national level at this stage.* |
| Objectives | 3 | State specific objectives, including any prespecified hypotheses  *-> Page 3, last paragraph before methods. “The purpose of this study was to describe secular trends in the proportion of older US adults reporting community/senior center meals at a national level using data from the US National Health and Nutrition Examination Surveys (NHANES) from 2009 to 2018. A special emphasis was put on identifying factors associated with community/senior center meal usage in older US adults.”* |
| Methods | | |
| Study design | 4 | Present key elements of study design early in the paper  *-> Page 4, methods section emphasizes the usage of complex, multistage, probability sampling design data from the cross-sectional National Health and Nutrition Examination Surveys (NHANES).*  *-> Page 4, paragraph 1, summarizes the NHANES: “The NHANES is a nationally representative, cross-sectional survey of the non-institutionalized, civilian US population with publicly available data (…)”* |
| Setting | 5 | Describe the setting, locations, and relevant dates, including periods of recruitment, exposure, follow-up, and data collection  *-> Pages 4 summarizes the setting (US), location (country-wide), relevant dates (NHANES 2009-2023) and period of recruitment, “exposure” (self-reported intake of community center meals), follow-up (n/a-> cross-sectional study), and data collection methods.* |
| Participants | 6 | (*a*) Give the eligibility criteria, and the sources and methods of selection of participants  *-> Page 4, section “Outcome”. Individuals with missing data on are variable of interest were excluded (see statistical analysis). A participant inclusion flow chart is provided for a better overview (eFigure 1).* |
| Variables | 7 | Clearly define all outcomes, exposures, predictors, potential confounders, and effect modifiers. Give diagnostic criteria, if applicable  *-> Page 4, section “Outcome”. Covariables and predictors are discussed in detail in the section “Covariables” on the same page.* |
| Data sources/ measurement | 8* | For each variable of interest, give sources of data and details of methods of assessment (measurement). Describe comparability of assessment methods if there is more than one group  *-> See above. The data source (NHANES) and its modules are described in detail.* |
| Bias | 9 | Describe any efforts to address potential sources of bias  *-> Self-reported data; mentioned here. Implications including recall bias are discussed later in the discussion section.* |
| Study size | 10 | Explain how the study size was arrived at  *-> eFigure 1 – participant inclusion flowchart* |
| Quantitative variables | 11 | Explain how quantitative variables were handled in the analyses. If applicable, describe which groupings were chosen and why  *-> All quantitative variables were handled as continuous variables (except income). Income was categorized in 3 groups (see Table 1) due to sample size considerations and the pre-existing NHANES data structure. The main outcome, intake of community /senior center meals, was a binary variable (yes/no).* |
| Statistical methods | 12 | (*a*) Describe all statistical methods, including those used to control for confounding  *-> Page 4, “Statistical Analysis”* |
|  |  | (*b*) Describe any methods used to examine subgroups and interactions  *-> Page 4, “Statistical Analysis”* |
|  |  | (*c*) Explain how missing data were addressed  *-> Participants without a complete dataset were excluded. No imputation procedures were performed.* |
|  |  | (*d*) If applicable, describe analytical methods taking account of sampling strategy  *-> Page 7. To account for the complex NHANES survey design characteristics and population weights, we performed weighted survey analyses throughout the entire analysis process using Stata’s “svyset” and “svy” commands.* |
|  |  | (*e*) Describe any sensitivity analyses  *-> Not performed.* |
| Results | | |
| Participants | 13* | (a) Report numbers of individuals at each stage of study—eg numbers potentially eligible, examined for eligibility, confirmed eligible, included in the study, completing follow-up, and analysed  *-> eFigure 1 – participant inclusion flowchart*  *-> The final sample included n = 7,808 unweighted observations, thereof n = 514 individuals reporting community/senior center meals.* |
|  |  | (b) Give reasons for non-participation at each stage  *-> see above in eFigure 1.* |
|  |  | (c) Consider use of a flow diagram  *-> eFigure 1 – participant inclusion flowchart* |
| Descriptive data | 14* | (a) Give characteristics of study participants (eg demographic, clinical, social) and information on exposures and potential confounders  *-> Table 1. Table 1 displays sociodemographic characteristics associated with the consumption of community/senior center meals.* |
|  |  | (b) Indicate number of participants with missing data for each variable of interest  *-> none, see above. Participants with missing data were not considered in this analysis.* |
| Outcome data | 15* | Report numbers of outcome events or summary measures  *Described in Figure 1.* |
| Main results | 16 | (*a*) Give unadjusted estimates and, if applicable, confounder-adjusted estimates and their precision (eg, 95% confidence interval). Make clear which confounders were adjusted for and why they were included  *See Figure 1. No adjustments were performed (secular trend analysis)* |
|  |  | (*b*) Report category boundaries when continuous variables were categorized  *Not applicable.* |
|  |  | (*c*) If relevant, consider translating estimates of relative risk into absolute risk for a meaningful time period  *Not applicable.* |
| Other analyses | 17 | Report other analyses done—eg analyses of subgroups and interactions, and sensitivity analyses  *Sub-group analyses were performed and visualized in Figure 1, panel C (by sex).* |
| Discussion | | |
| Key results | 18 | Summarise key results with reference to study objectives  *-> Page 8, paragraphs 1 and 2. “The herein analyzed data suggested a declining trend in senior/community center meals between 2009 and 2023 at the national level, corroborating previously suggested local trends [1].”* |
| Limitations | 19 | Discuss limitations of the study, taking into account sources of potential bias or imprecision. Discuss both direction and magnitude of any potential bias  *-> See page 9, first paragraph: “Geographical data (e.g., rural vs. city origin) was not available to us. Likewise, to maintain an adequate sample size, we could not consider other potentially important predictors (such as mental and physical health). Community center meals were self-reported and this information is thus susceptible to recall and reporting bias.* |
| Interpretation | 20 | Give a cautious overall interpretation of results considering objectives, limitations, multiplicity of analyses, results from similar studies, and other relevant evidence  *-> See last paragraph of the discussion. “Nevertheless, our study adds to the literature by re-emphasizing the importance of senior/community center meal programs and by highlighting that more than 2% of the older US population consistently report meal consumption at these focal care points.”* |
| Generalisability | 21 | Discuss the generalisability (external validity) of the study results  -> *Done in detail under consideration of the sample size, data structure and origin as well as with regard to the unique characteristics of the analysed NHANES data. The reliability of all weighted proportions were carefully assessed. Generalisability (and potential limitations) was discussed.* |
| Other information | | |
| Funding | 22 | Give the source of funding and the role of the funders for the present study and, if applicable, for the original study on which the present article is based  *The present study received no external funding.* |

*Give information separately for exposed and unexposed groups.

**Note:** An Explanation and Elaboration article discusses each checklist item and gives methodological background and published examples of transparent reporting. The STROBE checklist is best used in conjunction with this article (freely available on the Web sites of PLoS Medicine at http://www.plosmedicine.org/, Annals of Internal Medicine at http://www.annals.org/, and Epidemiology at http://www.epidem.com/). Information on the STROBE Initiative is available at www.strobe-statement.org.
